# Recent Synthetic Approaches Toward Non-anomeric Spiroketals in Natural Products 

**DOI:** 10.3390/molecules13102570

**Published:** 2008-10-17

**Authors:** Sylvain Favre, Pierre Vogel, Sandrine Gerber-Lemaire

**Affiliations:** Laboratory of Glycochemistry and Asymmetric Synthesis, Ecole Polytechnique Fédérale de Lausanne, Batochime, CH-1015 Lausanne, Switzerland

**Keywords:** Spiroketal, anomeric interaction, non-anomeric interaction, thermodynamic equilibration, kinetic control, spirocyclization, anomeric effect, gauche effect

## Abstract

Many natural products of biological interest contain [6,5]- and [6,6]-spiroketal moieties that can adopt various configurations, benefiting or not from anomeric conformation stabilizing effects. The spiroketal fragments are often important for the biological activity of the compounds containing them. Most stable spiroketal stereoisomers, including those benefiting from conformational anomeric effects (*gauche* conformers can be more stable than *anti* conformers because of a contra-steric stabilizing effect), are obtained easily under acidic conditions that permit acetal heterolysis (formation of tertiary oxycarbenium ion intermediates). The synthesis of less stable stereoisomers requires stereoselective acetal forming reactions that do not permit their equilibration with their most stable stereoisomers or, in the case of suitably substituted derivatives, concomitant reactions generating tricyclic products that quench the less stable spiroketal conformers. Ingenuous approaches have been recently developed for the synthesis of naturally occurring [6,6]- and [5,6]-nonanomeric spiroketals and analogues. The identification of several parameters that can influence the stereochemical outcome of spirocyclization processes has led to seminal improvements in the selective preparation of the non-anomeric isomers that are discussed herein. This review also gives an up-dated view of conformational anomeric effect which represents a small fraction of the enthalpic anomeric effect that makes *gem*-dioxy substituted compounds much more stable that their 1,n-dioxy substituted isomers (n > 1). Although models assuming sp^3^-hybridized oxygen atoms have been very popular (rabbit ears for the two non-bonding electron pairs of oxygen atom), sp^2^-hybridized oxygen atoms are used to describe the conformational anomeric effect.

## Introduction

**Figure 1:**
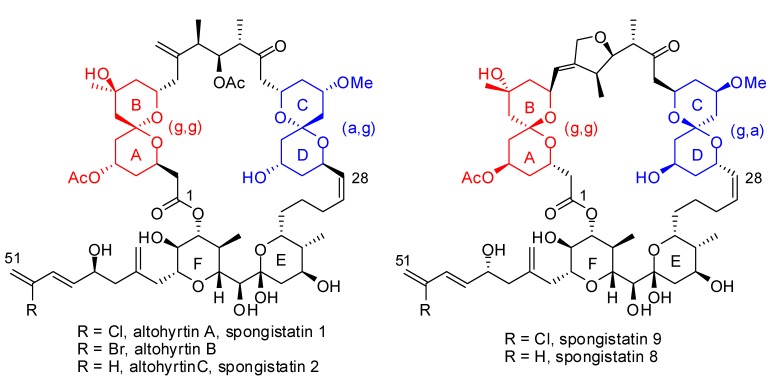
Members of the spongistatins/altohyrtins family.

Spiroketals and in particular 6,6-congeners are key structural features of a variety of natural products of biological interest which include marine macrolides, ionophores and polyether antibiotics [1]. For instance, spongistatins (altohyrtins), which were isolated from marine sponges of the genus *Spongia* by three research groups in 1993, present two highly functionalized 6,6-spiroketal subunits in their skeleton (Figure 1) [2]. Interestingly, while the AB spiroketal adopts a (*gauche*, *gauche*)-conformation and thus benefits from a double conformational anomeric effect (two axial C-O moieties in chair tetrahydropyran rings), the CD spiroketal stands in a (*anti*, *gauche*)-conformation benefiting from a single anomeric effect (one C-O of one tetrahydropyran ring in axial position with respect to the other ring). Most of the methodologies that have been developed for the synthesis of spiroketals are based on acid catalyzed spirocyclization of dihydroxy-ketone precursors which generally lead to the most stable stereoisomers and conformers. The obtention of the less stable spiroketals (*gauche*, *anti* or *anti*, *anti*) requires either the reversal of the thermodynamic stability by additional stabilizing interactions such as hydrogen bonding or a kinetic control for the formation of the spiroketal. This review will highlight the recent strategies [3] that have been developed for the selective synthesis of spiroketals in natural products, with particular emphasis on the AB and CD subunits of spongistatins / altohyrtins.

## 2. Relative stability of spiroketal stereoisomers and conformers

**Figure 2:**
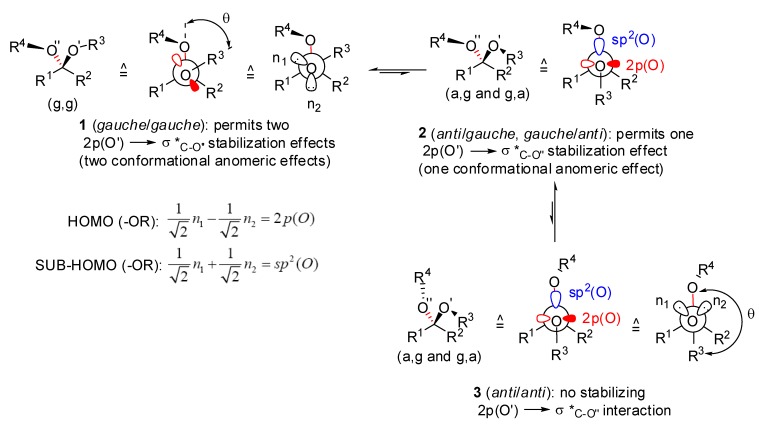
Possible conformations of hemiacetals and acetals.

Acetals are more stable than their 1,n-dialkoxyalkane isomers (n>1). Examples are given by comparing the standard heats of formation (gas phase) of 1,2-diethoxyethane (ΔH_f_° = -98.0 kcal/mol) with that of 1,1-diethoxyethane (ΔH_f_° = -108.4 kcal/mol), of 1,3-diethoxypropane (ΔH_f_° = -104.3 kcal/mol) with that of 2,2-diethoxypropane (ΔH_f_° = -121.1 kcal/mol), or of 1,4-dioxane (ΔH_f_° = -75.4 kcal/mol) with that of 1,3-dioxane (ΔH_f_° = -80.9 kcal/mol). The *gem*-dioxy substitution stabilizing effect is called the “enthalpic anomeric effect” [4]. Known for alkyl pyranosides since 1905 (e.g.: alkyl α-d-glucopyranosides being more stable than their β-anomers) [5] and rediscovered in 1955 by Edward [6], and by Lemieux and Chü in 1958 [7], the conformational anomeric effect designs the contra-steric effect observed in acetals which renders the more sterically encumbered *gauche*/*gauche* conformers (**1**) more stable than their *anti*/*gauche* (**2**) and *anti*/*anti* (**3**) conformers (Figure 2). [IUPAC defines prefix *anti* (antiperiplanar) for two vicinal σ bonds making a dihedral angle 150 < θ < 210°, and prefix *gauche* for two vicinal σ bonds making a dihedral angle 30 < θ < 90°].

The conformational anomeric effect explains the higher stability of alkyl α-d-glucopyranosides with respect to their β-anomers and the *exo*-anomeric effect in these compounds [8]. It has been ascribed to σ conjugation [9] of the type 2p(O’)→σ*_C-O’’_ (negative hyperconjugation).^10^ Similar effects were proposed by Deslongchamps and co-workers to intervene on the stability difference of 1,7-dioxaspiro[5.5]undecanes (6,6-spiroketals) [11]. Differences in stability between acetal conformers (or stereoisomers) benefiting from possible hyperconjugation interactions 2p(O’)→σ*_C-O’’_ and those in which these stabilizing effects are prohibited by their geometry (2p(O’) orbital perpendicular to the empty σ*_C-O’’_ orbital) is only a fraction (-1 to -3 kcal/mol) of the global enthalpic anomeric effect or *gem*-dioxy stabilizing effect (-6 to -17 kcal/mol). Dipole/dipole stabilizing interactions intervene also in hemiacetals, acetals and ketals (acetals derived from ketones). Depending on substitution, steric factors can affect the relative stability of acetal conformers [4c,12]. Differences in stability between acetal conformers of type **1**, **2** and **3** are also solvent dependent [13]. For instance, 2-hydroxytetra-hydropyran prefers an axial hydroxyl group in the gas phase and in non polar solvents [14] whereas it prefers an equatorial hydroxyl group in aqueous solution [15]. In many instances, a sp^3^-hybridized oxygen atom is used to describe the properties of acetals (with two sp^3^ orbitals occupied each by two electrons pairs: rabbit ears). This model is reminiscent of the banana bonds model used sometimes to interpret properties of π systems such as alkenes and carbonyl moieties. Electron distribution about oxygen atom in H_2_O [16], ethers and related compounds are better represented by using a sp^2^-hybridized oxygen atom [17]. In fact sp^2^(O), the SUB-HOMO is equivalent to the combination 1/√2 sp^3^(O) + 1/√2 sp^3^(O) and the HOMO, localized on the ethereal oxygen atom is equivalent to 1/√2 sp^3^(O) - 1/√2 sp^3^(O). As the latter is higher in energy than the former localized orbital, it contributes more efficiently to any electron transfer, for instance into a hyperconjugated C-X bond. We thus prefer to use sp^2^-hybridized than sp^3^-hybridized oxygen atoms in spiroacetals. Moreover, the analysis of the crystal structures of [6,6]-spiroketal derivatives recorded in the Cambridge Structural Database revealed that the dihedral angle θ (Figure 2) defined by the O-C, O-C bonds of the spiroketals is larger than 60° in most cases (mean value: 62° for 84 [6,6]-(*gauche*/*gauche*)-spiroketal structures). This observation is in agreement with the sp^2^(O) model depicted in Figure 2 for the conformational anomeric effect which intends to optimize overlap between 2p(O) (HOMO) and σ*_C-O_ (LUMO) orbitals . Recent quantum calculations and topological analysis using the Quantum Theory of Atoms in Molecules (QTAIM) [16] provide an explanation of the conformational anomeric effect for acetals that is not in line with the n(O) →σ*_C-O_ hyperconjugation model. The conformational energy variations are accompanied by an electron population redistribution that implies the CH_2_ group in MeOCH_2_OMe, and by extension the CR^1^R^2^ group in other acetals. The stabilization of the *gauche* conformers of (MeO)_2_CH_2_ is accompanied by a progressive reduction of the electron population of the hydrogens of the central CH_2_ group as the number of their gauche interactions to lone pairs rises. The electron population removed from these atoms during the conformational change is gained in the *gauche* conformers by the oxygen atoms, which results in more negative energy. The electrostatic effects (hard interactions) thus overwhelm the polarizability effect (hyperconjugation: soft interactions) [18]. In the case of acetals with vicinal oxy or other polar substituents, attractive gauche effects [19] might also contribute to the difference in stability of the acetals conformers or/and stereoisomers [20]. 

**Figure 3:**
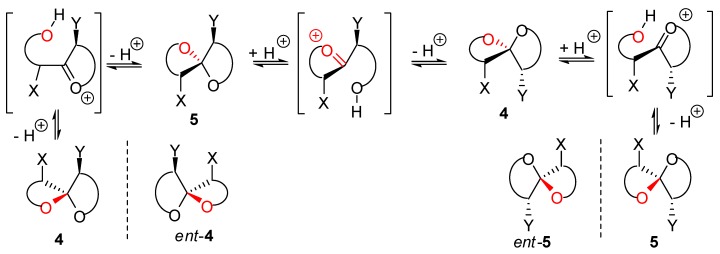
Epimerization of spiroacetals through acid-catalyzed heterolysis.

Unsubstituted [n,n]-spiroketals have a C_2_ axis – they are chiral. Unsubstituted [n,m]-spiroketals with n≠m are also chiral (C_1_ symmetry). Thus on substituting these systems with one, two, …substituents X, Y, … one, two, …pairs of diastereoisomers can be generated. This is illustrated in Figure 3 for disubstituted spiroketals. Heterolysis of the acetal moiety allows epimerization of the anomeric center but does not racemize these systems. In the case of **4**, acetal epimerization give **5** whichever C-O bond undergoes heterolysis.

**Figure 4:**
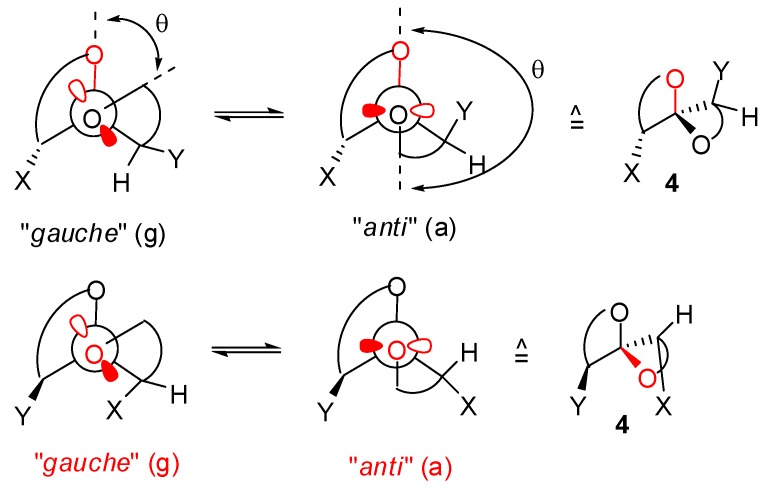
Possible conformers for diastereoisomeric [n,m]-spiroketals (n,m > 3).

Except for [3,3]-spiroketals that can adopt only one conformation because of the planar oxirane moieties, the other spiroketals with oxetane, tetrahydrofuran and tetrahydropyran rings can generate various conformers (puckering in oxetane [21], twist and envelop conformers for tetrahydrofurans [22], chair and twist boat conformers for tetrahydropyrans [23]) as shown in Figure 4. Depending on the nature of substituents X and Y, the four possible conformations (g, 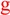), (g,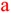), (a,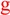) and (a,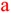) shown for **4** might have different relative stabilities. Ignoring differential solvation effects, steric factors, gauche effects, electrostatic effects and intramolecular hydrogen bridging due to substituents X and Y, one expects that (g,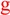)-conformers are more stable than both (g,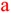)- and (a,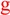)-conformers, themselves expected to be more stable than the corresponding (a,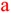)-conformers for stereoelectronic reasons (see above). In the cases of [5,6]- and [6,6]-spiroketals, the tetrahydropyran ring usually prefers chair conformations. Their interconversions are not degenerate upon substitution as equatorial substituents in one chair occupy axial positions (destabilizing gauche interactions) in the other. This might affect stability difference between (g,g), (g,a), (a,g) and (a,a)-conformers and favor one of the two possible diastereoisomeric spiroketals (**4** *vs* **5**), should they equilibrate by acetal epimerization (Figure 3). If substituents X and Y are part of a bridge in a tricyclic structure, any of the (g,g), (g,a), (a,a) or (a,a) conformations might be obtained by the adequate choice of the nature of the bridge and relative configuration of the substituted centers. Such bridging might also favor other conformations than chairs for their tetrahydropyran moieties.

Most natural products containing spiroketal subunits display five and six membered rings. The pioneering synthetic efforts devoted to the preparation of such structures assumed that the configuration of the spiro carbon in natural compounds corresponds to the most stable (g,g)-conformation. In fact, several naturally occurring spiroketals adopt other conformations. This review covers the recent synthetic approaches to naturally occurring non-anomeric [6,6]- and [6,5]-spiroketals with particular focus on the spiroketal fragments of spongistatins/altohyrtins.

## 3. Synthesis of naturally occurring [6,6]-spiroketals

### 3.1. AB and CD spiroketals of spongistatins/altohyrtins

In 1993, the research groups of Pettit, Kitagawa/Kobayashi and Fusetani independently reported the isolation, from marine sponges, of a family of related highly oxygenated macrolactones containing highly substituted 6,6-spiroketals and tetrahydropyran rings [24]. These molecules were found to be among the most potent cancer cells growth inhibitors tested to date by the U.S. National Cancer Institute on a panel of 60 human carcinoma cell lines, with GI_50_ values in the nanomolar and picomolar ranges. Despite these promising properties, further biological investigations were precluded by the extremely low quantities that can be obtained by extraction of marine organisms and the unacceptable ecological impact of larger scale isolation of the producing sponges. This scarce abundance, combined with remarkable structural complexity, prompted many research groups to address the challenge of the synthesis of these macrolides. The structure of these appealing molecules is based on a 42-membered macrolactone embedded with two spiroketal subunits (AB and CD) and two highly substituted tetrahydropyrans (E and F), and functionalized by a sensitive trienic side chain, for a total of 24 stereocenters. Spongistatins 5, 7-9 contain an additional 5 membered ring within the skeleton. Interestingly, while the AB spiroketal adopt a (g,g)-conformation, the CD spiroketal stands in a (g,a)-conformation. It probably gains some stabilization from intramolecular hydrogen bonding. The routes developed, after 2004, toward the obtention of the (g,a)-CD subunit *vs* the (g,g)-AB subunit of these macrolides will be detailed.

**Scheme 1:**
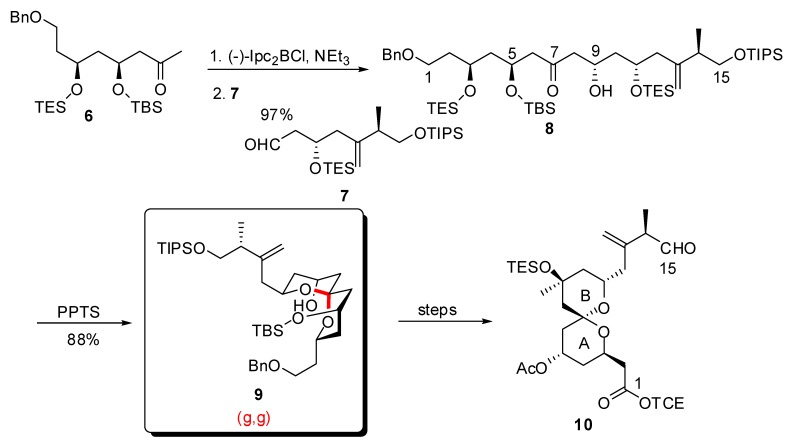
Synthesis of the AB spiroketal subunit of spongistatin1 by Paterson *et al*.

After a first report on the total synthesis of spongistatin 1/altohyrtin A in 2001 [25], the Paterson group disclosed more recently a detailed account of their studies on this target [26]. Approach to the C_1_-C_15_ AB spiroketal subunit was based on acid-catalyzed acetal formation from a linear precursor to deliver the thermodynamically favored isomer (Scheme 1) [26a]. The dihydroxy ketone precursor **8** arose from addition of the boron enolate from methyl ketone **6** to aldehyde **7**, thus installing the 5,9-*anti* relationship of the alcohols. Selective removal of the triethylsilyl groups in the presence of PPTS induced spirocyclization to the unique thermodynamically favoured spiroketal **9**. This intermediate was, in turn, efficiently transformed into the fully elaborated AB fragment of spongistatin 1/altohyrtin A.

**Scheme 2:**
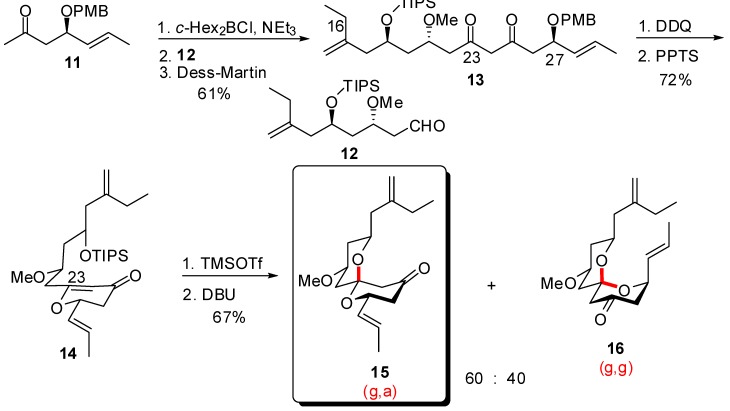
Kinetically-controlled synthesis of the CD spiroketal of spongistatin 1 according to Paterson *et al*.

For the synthesis of the CD fragment, a stepwise construction of both rings was envisaged through a kinetically-controlled intramolecular hetero-Michael addition on a D-ring dihydropyrone precursor (Scheme 2) [26b]. 

Stereoselective boron aldol coupling to aldehyde **12** and subsequent oxidation of the intermediate aldol at C(23) provided the precursor of ring D. Removal of the PMB ether, followed by dehydrative cyclization afforded dihydropyranone **14** in good yield. Desilylation allowed intramolecular hetero-Michael addition on enone at C(23) position, under mild basic conditions. Nevertheless, the desired, less stable, (g,a)-spiroketal **15** was obtained with modest selectivity, along with the undesired (g,g)-spiroketal **16**.

The same group reported another route to the CD spiroketal of spongistatin 1, through thermodynamic control [26b]. Aldol condensation of boron enolate derived from methyl ketone **17** to aldehyde **18** ensured the *anti* relationship of hydroxyls at C(21) and C(25). Acidic cleavage of silyl ethers led to spirocyclization to provide a 1:5 mixture of the desired (g,a)-spiroketal **21** and the undesired thermodynamically favoured (g,g)-isomer **20**. Several cycles of equilibration assisted by hydrogen bonding / separation provided larger quantities of the desired spiroketal, up to 69% yield. Separation of the two isomers by gradient elution column chromatography delivered gram scale quantity of the (g,a)-spiroketal **21**. Further functionalization generated the CD spiroketal **22** of spongistatin 1.

**Scheme 3:**
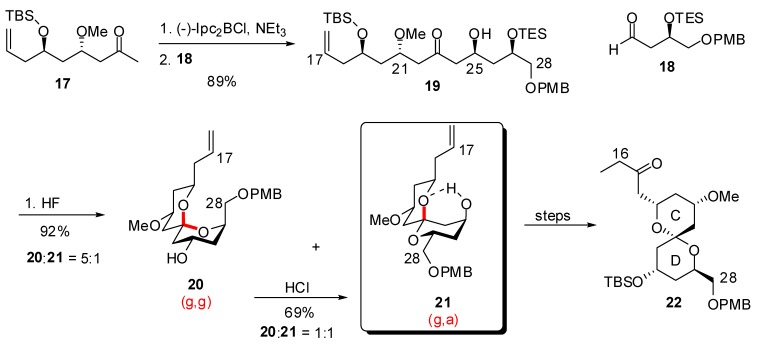
Synthesis of the CD spiroketals of spongistatin 1 under thermodynamic control according to Paterson *et al*.

While both approaches generated a mixture of spiroacetal epimers, the second route using thermodynamically controlled spirocyclization followed by acid-catalyzed equilibration was practically more useful due to easier separation of spiroketals **20** and **21**.

**Scheme 4:**
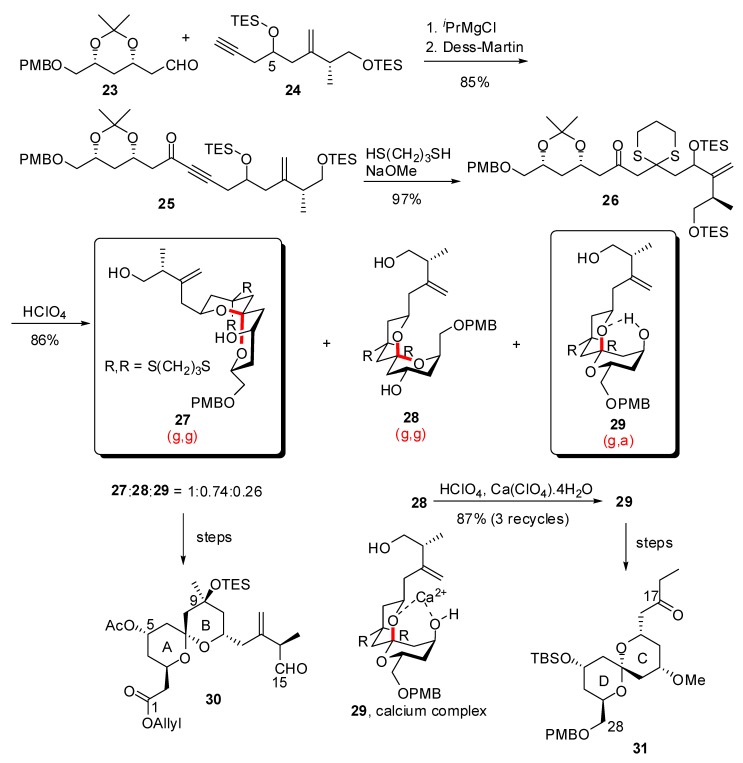
Synthesis of AB and CD spiroketals of spongistatin 1 according to Ley *et al*.

Guided by the latent *C*_2_ pseudo-symmetry of spongistatin 1 about the C(15) atom, Ley and co-workers designed a synthetic pathway where both AB and CD spiroketals derive from a common linear precursor [27]. For that purpose, addition of the acetylide anion of a 1:1 C(5) epimeric mixture **24** to aldehyde **23**, followed by oxidation of the intermediate alcohol led to ynone **25** (Scheme 4). Double conjugate addition of 1,3-propanedithiol and subsequent acidic treatment provided a 1:0.7:0.26 mixture of three spiroketals (**27**, **28**, **29**) presenting the AB (g,g)-, CD (g,g)- and CD (g,a)- arrangements, respectively. Successive equilibration protocols in the presence of Ca^2+^ / perchloric acid efficiently converted the undesired (g,g) spiroketal **28** into its (g,a) isomer **29**. These intermediates were then converted into fully elaborated AB and CD spiroketals of spongistatin 1 (**30**, **31**). Interestingly, this methodology took advantage of the latent *C*_2_ pseudo-symmetry of spongistatin 1 about the C(15) atom to give a rapid access to both AB and CD spiroketals from the same linear precursor. Furthermore, in the scale-up synthesis of spongistatin 1, the same group reported a highly efficient azeotropic reflux chromatography to separate the isomeric spiroketals **27**-**29** [28].

**Scheme 5:**
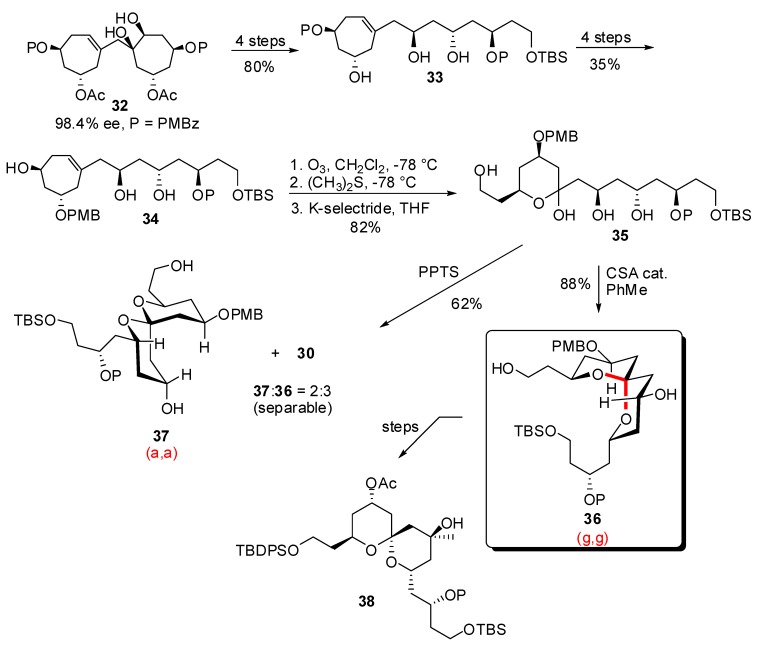
Synthesis of an advanced precursor of the AB spiroketal of spongistatins according to Vogel *et al*.

Based on the methodology previously developed for the asymmetric synthesis of C_15_ polyketides from readily available 2,2’-methylene difuran, Vogel *et al*. disclosed an efficient pathway to an advanced precursor of the AB spiroketal of spongistatins, and analogues (Scheme 5) [29]. Functionalization of the enantiomerically enriched diol **32** led to semi-opened polyol **33**. Further orthogonal protecting group manipulations led to the key intermediate olefin **34**. A sequence of ozonolysis / reductive treatment afforded hemiketal **35** in high yield. Acidic treatment with CSA allowed spirocyclization to the thermodynamic (g,g)-spiroketal **36** which was further transformed into and advanced precursor of the AB spiroketal of spongistatins **38**. Interestingly, when **35** was submitted to PPTS, the cyclization process afforded a mixture of the non-anomeric spiroketal **37** ((a,a)-isomer) and the thermodynamic stereoisomer **36** in a 2:3 ratio. Variation on the nature of the acidic partner can therefore lead to different stereoisomeric 6,6-spiroketal from the same precursor.

**Scheme 6:**
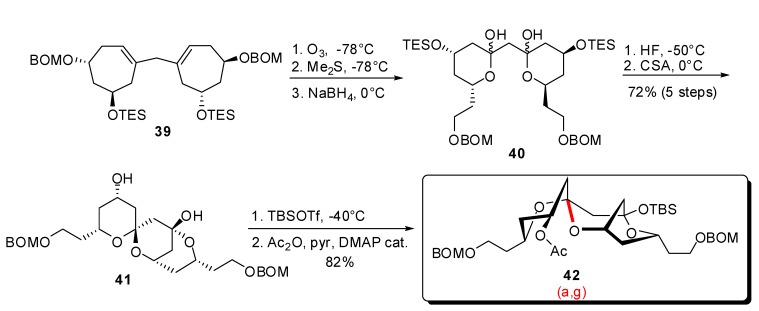
Synthesis of tricyclic spiroketals according to Vogel *et al*.

Following another approach based on the simultaneous functionalization of diolefin **39**, the same authors reported a sequence of ozonolysis / reductive treatment to afford bis-hemiketal **40** (Scheme 6) [30]. Subsequent acidic treatment, first with aqueous HF and then with CSA, induced spirocyclization to the tricyclic intermediate **41**, in high yield. 

The formation of a tricyclic structure allowed trapping of the kinetic (a,g)-spiroketal (one axial C-O bond for the [6,6]-spiroketal unit) as demonstrated by 2D-NMR experiments on the orthogonally protected derivative **42**.

### 3.2. Synthesis of other nonanomeric [6,6]-spiroketals related to natural products

**Scheme 7:**
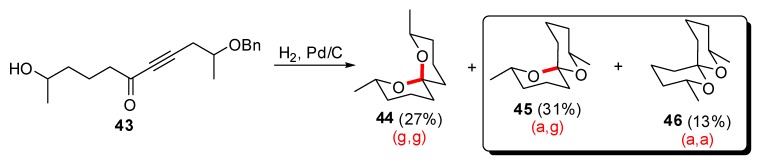
Access to thermodynamically non stabilized [6,6]-spiroketals according to Koutek *et al*.

In order to access spiroketals that do not adopt (g,g)-conformation (with two axial C-O bonds for their spiroketal moieties), several kinetic spirocyclization reactions have been developed. Mild non-acidic conditions, involving reduction of an ynone precursor, have been reported by Koutek *et al*. (Scheme 7) [31]. Hydrogenation of derivative **43** induced both reduction of the triple bond and hydrogenolysis of the benzyl ether to afford a mixture of isomeric spiroketals from which the “non-anomeric” derivatives **45** (axial/equatorial C-O bonds) and **46** (equatorial/equatorial C-O bonds) were predominant.

**Scheme 8:**
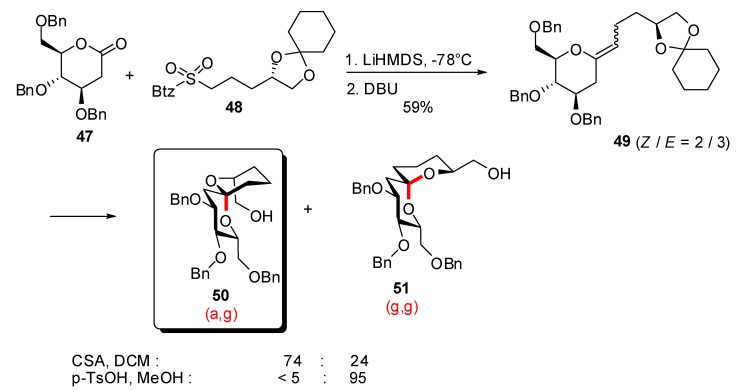
Access to “non-anomeric” spiroketals from carbohydrate derived precursors according to Goekjian *et al*.

Spirocyclization reactions from carbohydrate-derived precursors were also explored to access non-thermodynamic spiroketals. The group of Goekjian [32] prepared the *exo*-glycal derivative **49** through a modified Julia olefination on carbohydrate lactone **47** (Scheme 8). Treatment under kinetic conditions resulted in an initial 3.3:1 selectivity in favor of the (a,g)-spiroketal **50**. In contrast, the (g,g)-isomer **51** was obtained in the presence of p-toluene sulfonic acid.

**Scheme 9:**
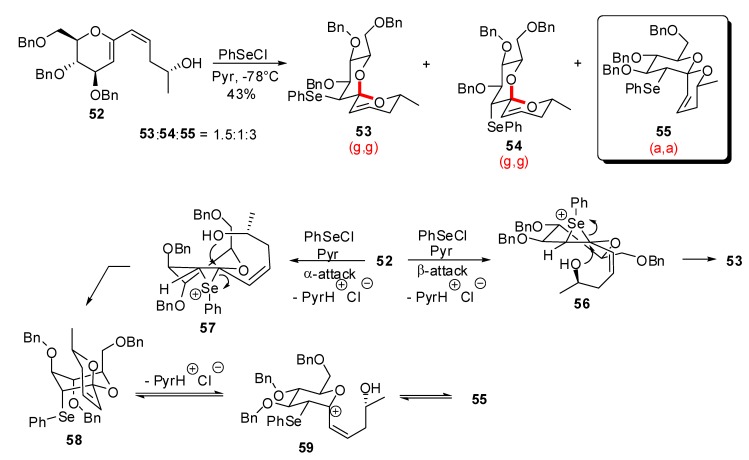
Carbohydrate-based synthesis of spiroketals according to Quayle *et al*.

A carbohydrate template was also disclosed by Quayle *et al*. [33] for the preparation of unsaturated spiroketals related to the milbemycins / avermectins family (Scheme 9). 

Exposure of dienol **52** to PhSeCl in basic medium promoted cyclization under kinetic control to deliver a mixture of spiroketals **53**-**55**, enriched in the non-anomeric (a,a)-isomer **55**. The major products probably arose from the *trans*-diaxial opening of kinetic *epi*-selenonium intermediates **56** and **57**. Formation of **55** would arise from intermediate **58** that undergoes facile isomerization via an allylic glycosyl cation intermediate **59**.

**Scheme 10:**
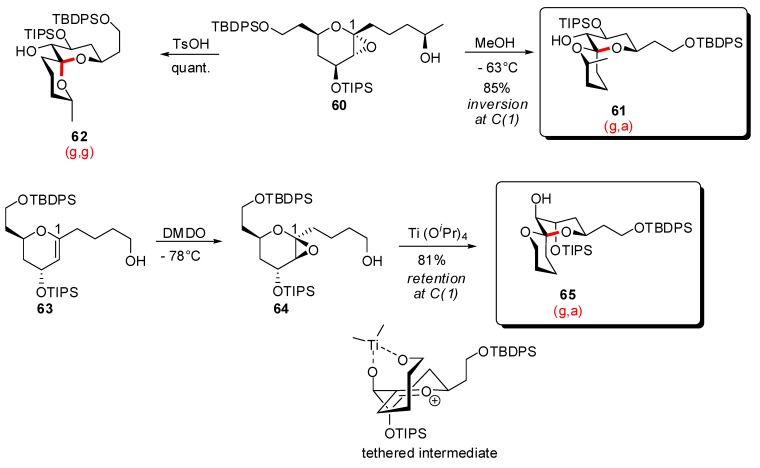
Spirocyclizations from glycal epoxides according to Tan *et al*.

Kinetic spirocyclization reactions were also disclosed by Tan *et al*. [34] through the sterocontrolled opening of glycal epoxides (Scheme 10). Treatment of epoxy-glycal **60** in the presence of MeOH at low temperature, induced equatorial installation of the oxygen nucleophile to deliver the (g,a)-spiroketal **61** with complete stereoselectivity and high yield. The authors suggested that the reaction proceeded through MeOH hydrogen bonding catalysis. The corresponding (g,g)-spiroketal **62** was isolated in excellent yield after subjection of the same starting epoxide to acidic conditions. The same research group similarly explored the reactivity of stereoisomeric glycal epoxides such as **64**, formed *in situ* by epoxidation of *erythro*-glycal **63** [35]. Treatment of the nascent epoxide with multidentate Lewis acids, in particular Ti(O*^i^*Pr)_4_, induced spirocyclization with retention of configuration at C(1) to produce the (g,a)-spiroketal **65**, through a metal chelated early transition state. These methodologies were extended to other related glycal epoxides, thus giving access to stereochemically diversified spiroketals.

**Scheme 11:**
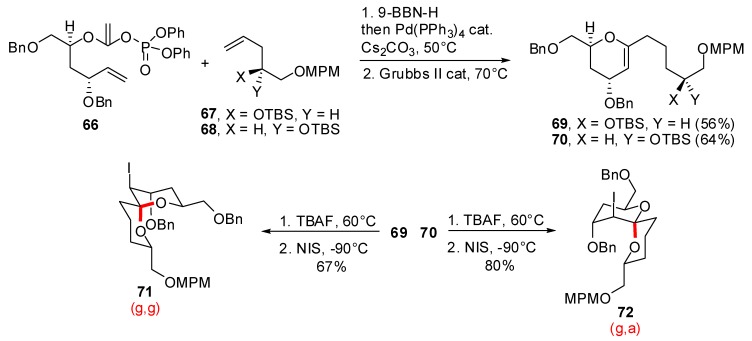
Synthesis of spiroketals from endocyclic enol ethers according to Fuwa and Sasaki.

Fuwa and Sasaki reported the use of endocyclic enol ethers in kinetically controlled spirocyclizations to access both anomeric and non-anomeric spiroketals (Scheme 11) [36]. The precursor enol ethers were synthesized through a Suzuki-Miyaura coupling / ring closing metathesis sequence from enol phosphates of type **66**. Depending on the local structure of the enol ether (configuration of the alcohol moiety), iodospirocyclization led either to the anomeric (g,g)-spiroketal **71** or to the non-anomeric (a,g)-spiroketal **72**, with high stereoselectivities.

**Scheme 12:**
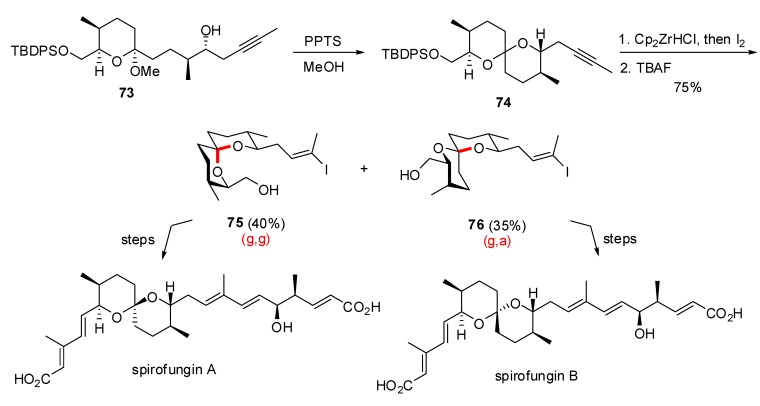
Synthesis of the spiroketals of Spirofungins A and B according to Shimizu *et al*.

Several reports also indicated the use of an initial mixture of both anomeric (g,g)- and non-anomeric (a,g)- or (g,a)-spiroketals to access stereochemically related natural products from a common precursor. In their synthetic studies toward the antifungal antibiotics spirofungins, Shimizu *et al* [37] proposed a spiroketalization process induced by PPTS on alkynyl methyl ketal **73** to produce a mixture of epimeric spiroketals **74** (Scheme 12). 

Functionalization of the triple bond and removal of the silyl ether allowed separation of the (g,g)-spiroketal **75** and the (g,a)-stereoisomer **76** in 40 and 35% yield, respectively. These two intermediates were engaged in the same synthetic sequence to deliver spirofungin A and spirofungin B in good overall yields. The stereochemically related pteridic acids A and B were also prepared from a common precursor by Paterson *et al*. [38]. Smooth silyl ether cleavage on enone **70** afforded a roughly equimolar mixture of spiroketals **71** and **72** presenting axial / axial and axial / equatorial configurations, respectively. Separation of these isomers provided access to pteridic acids A and B through a common synthetic sequence.

**Scheme 13:**
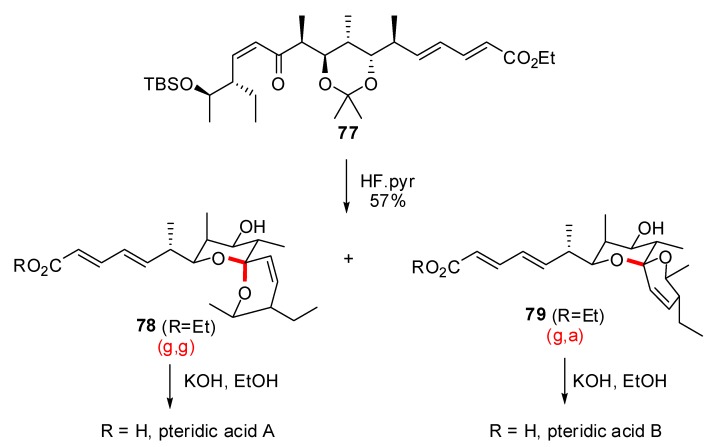
Synthesis of the spiroketals of Spirofungins A and B according to Paterson *et al*.

**Scheme 14:**
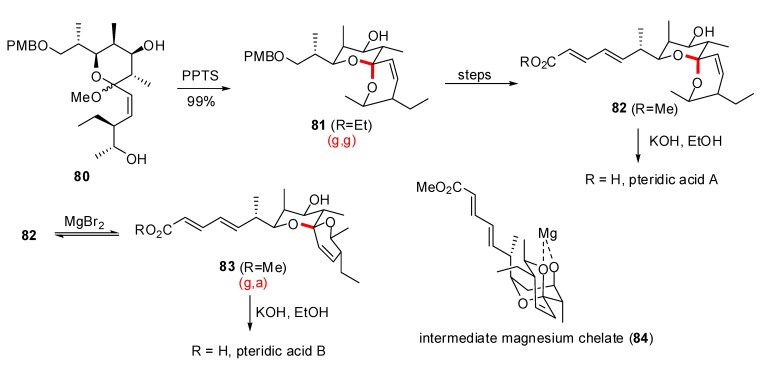
Synthesis of the spiroketals of pteridic acids A and B according to Kuwahara *et al*.

Another approach to pteridic acids A and B was proposed by Kuwahara *et al*. (Scheme 14) [39]. The (g,g)-spiroketal of pteridic acid A was obtained through acidic treatment of the methyl ketal precursor **80**, resulting in the selective formation of the (g,g)-isomer. Subsequent transformation provided methyl ester **82** as the direct precursor of pteridic acid A. Lewis acid catalyzed epimerization of **82** allowed the isolation of the anomerically disfavored spiroketal **83** in 40% yield, through the magnesium chelate intermediate **84**. A final hydrolysis led to pteridic acid B.

## 4. Synthesis of naturally occurring nonanomeric [6,5]-spiroketals

While [6,6]-spiroketal systems are the most common nonanomeric spiroketals in natural products, the nonanomeric [6,5]-spiroketal core is also present in many natural products of biological interest and thus lead to important synthetic efforts.

**Scheme 15:**
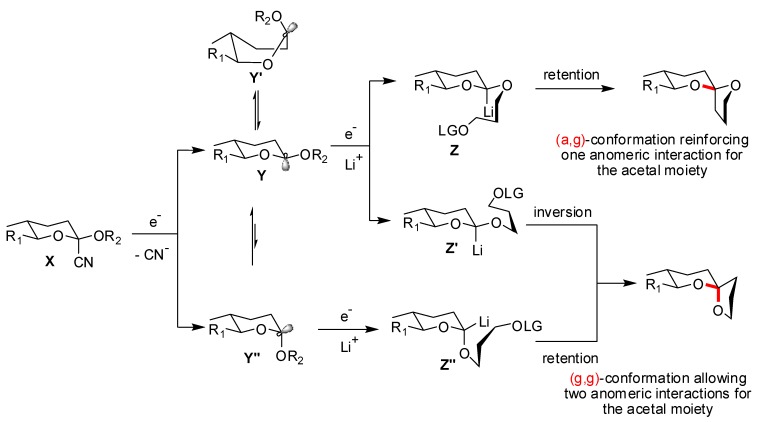
Mechanism of cyclization according to Rychnovsky *et al*.

Rychnovsky and co-workers took advantage of their methodology developed for the synthesis of non-anomeric [5,6] and [6,6]-spiroketals [40] to the synthesis of the AB-spiroketal of Pectenotoxin 2 [41] and to Attenol A [42]. By reversing the usual roles of electrophile and nucleophile in the synthesis of spiroketals, the preferred anomeric isomer was efficiently suppressed. Treatment of cyanoacetal **X** by lithium di-tert-butylbiphenylide (LiDBB) furnished, through single electron transfer and after the loss of the cyanide anion, the anomeric radical **Y** (Scheme 15) [43]. When the second electron was added during the reduction, the resulting alkyl lithium species **Z** remained locked in the axial position [44]. If the electrophilic substitution occured with retention of configuration, the (a,g)-spiroketal was generated. Competitive pathways, reaction of the equatorial alkyllithium species **Z’’** or inversion during the substitution of **Z’**, explained the formation of the minor (g,g)-epimer.

**Scheme 16:**
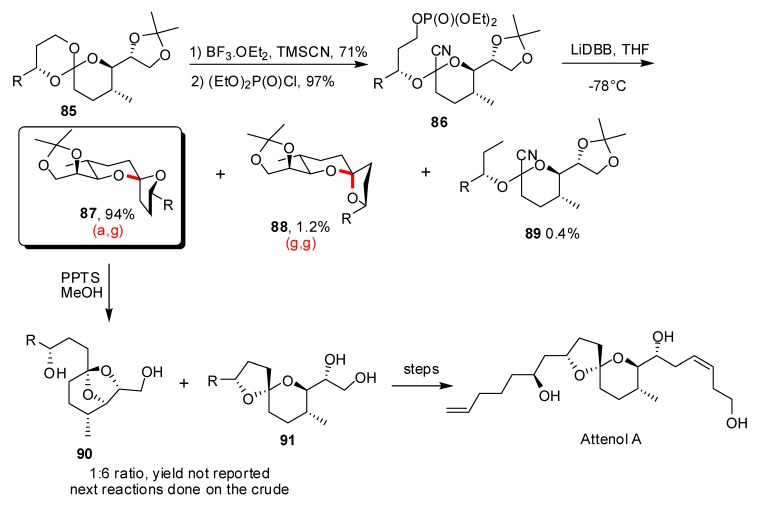
Synthesis of the spiroketal core of Attenol A according to Rychnovsky *et al*.

In their non-traditional approach to Attenol A [45] the Rychnovsky group synthesised the nonanomeric epimer of the natural product and then epimerized it to obtain the targeted molecule. This was more efficient than previously developed synthesis and demonstrates the potential of using reductive cyclisation reactions to afford non-anomeric spiroketals as well as their thermodynamically stabilized epimers (Scheme 16). After the elaboration of a practical route to spiro-orthoester **85**, treatment with TMSCN and BF_3_·OEt_2_ furnished the corresponding alcohol, with high regio and chemoselectivity, in 71% yield. Substitution of the primary alcohol by a phosphonate ester leaving group occurred under mild conditions to afford **86** in 97% yield. After addition of LiDBB, the reductive cyclization occurred at –78°C to deliver the (a,g)-spiroketal **87** as the major product in a remarkable 94% yield. The minor products were identified as the (g,g)-epimer and cyanoacetal **89** resulting from the competitive reduction of the phosphate group. GC analysis of the crude mixture established a 78:1 ratio of (a,g)- to (g,g)-spiroketal underlining the potential of this powerful approach. Equilibration toward the configuration of the natural product occurred in methanol, in the presence of PPTS and provided, after cleavage of protecting groups, the bicyclic derivative **90** together with spiroketal **91**. Attempts to suppress this competitive cyclization were unsuccessful and the mixture was used in the next reactions to complete the total synthesis of Attenol A.

**Scheme 17:**
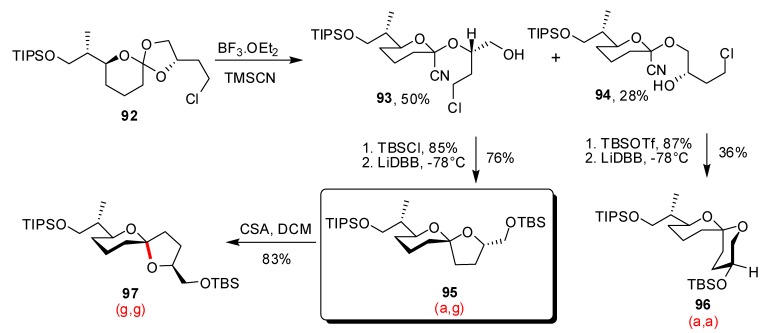
Synthesis of the spiroketal core of pectenotoxins according to Rychnovsky *et al*.

In 2007, the (a,g)-[5,6]-spiroketal core of pectenotoxins PTX1-3 and PTX6 was obtained by Rychnovsky and co-workers using the same methodology [46]. In this case, displacement of spiro-orthoesther **92** by TMSCN afforded two regioisomers **93** and **94** in 50% and 28% yield, respectively (Scheme 17). This lack of selectivity was attributed to the fact that **92** was less hindered than the previously studied intermediates. Treatment of the major cyanoacetal with LiDBB at low temperature furnished the (a,g)-spiroketal as a single isomer, in 76% yield. 

Epimerization of **95** under acidic conditions was also possible in this case and delivered the energetically favoured (g,g)-spiroketal **97** in 83% yield, corresponding to spiroketal core of other members of the pectenotoxins family (PTX4 and PTX7). Valuation of the minor cyanoacetal **94** was realised by its conversion into the (a,a)-[6,6]-spiroketal **96** (two C-O bonds of the spiroketal unit occupying equatorial positions) in a modest but non-optimized 36% yield, thus extending the scope of this methodology.

**Scheme 18:**
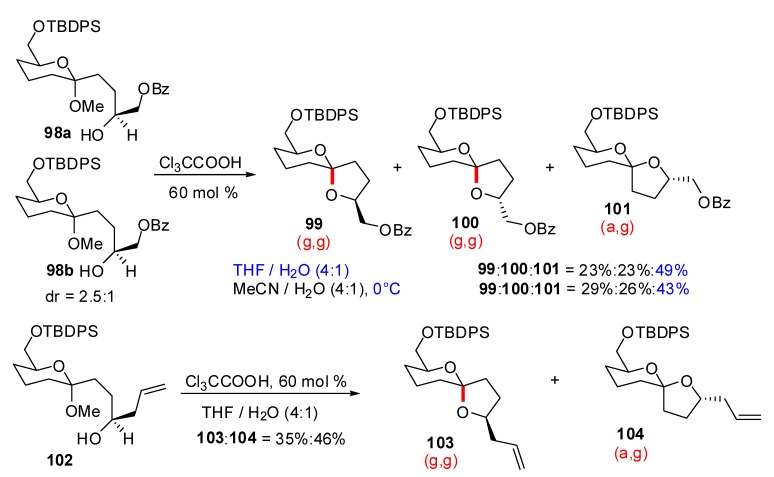
Synthesis of nonanomeric [5,6]-spiroketals according to Pihko *et al*.

Following their synthesis of the nonanomeric (a,g)-spiroketal core of the pectenotoxins [47] the group of Pihko further explored the preparation of nonanomeric [6,5]-spiroketals through kinetic control in acid catalyzed spirocyclization reactions [48]. Treatment of mixed ketal-alcohols under acid catalysis led to the formation of the nonanomeric spiroketals if the acid was carefully tuned and in aqueous THF medium (Scheme 18).

In particular, treatment of a mixture of methyl ketals **98a** and **98b**, in the presence of trichloroacetic acid, led the nonanomeric (a,g)-spiroketal **101** as the major isomer if the reaction was carried out in aqueous THF. Non aqueous conditions predominantly led to the formation of the anomeric isomers **99** and **100**. In the presence of acetonitrile, the nonanomeric spiroketal **101** could also be obtained in good yield by decreasing the temperature to 0°C. The above conditions have also been successfully applied to the allyl-substituted precursor **102** to deliver the spiroketals **103** and **104** in 35 and 46% yield, respectively.

**Scheme 19:**
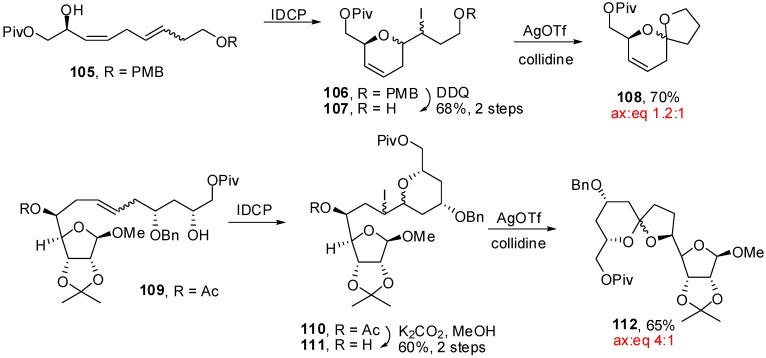

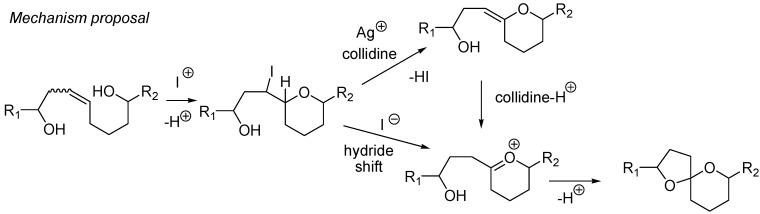
Synthesis of spiroketals according to Mootoo *et al*.

Another strategy was developed by the group of Mootoo [49] to produce spiroketals of various sizes and configurations through a iodoetherification/dehydroiodination sequence (Scheme 19). 

The starting hydroxyalkenes, obtained through cross-metathesis, were treated with iodonium dicollidine perchlorate (IDCP) to provide mixtures of diastereoisomeric iodinated intermediates **106** and **110**. The NMR data did not allow for distinction between the possible regioisomers but their structures were tentatively assigned to the 5-*exo*-trig cyclized products based on previous investigations [50]. Exposure of these iodo-alkenes to silver triflate in the presence of collidine led to the formation of [5,6]-spiroketals in moderate to good yields, with various ratios of (a,g)- and (g,g)-stereoisomers. The authors observed that the stereoselectivity of the spiroketalisation was not affected by the stereochemistry of the iodinated precursor which is consistent with an oxycarbenium ion mediated mechanism. They proposed two pathways that could lead to this intermediate: i) either protonation of an initially formed exocyclic enol ether (resulting from β-elimination of HI) or ii) via Ag^+^ assisted iodide S_N_1 departure with simultaneous hydride shift from the iodo ether intermediate.

**Scheme 20:**
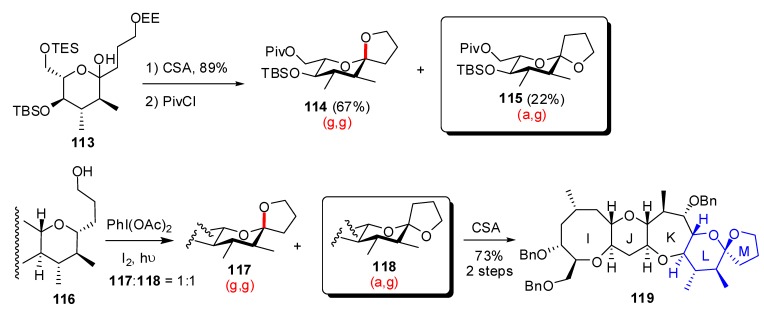
Synthesis of the LM spiroketal of ciguatoxin CTX3C according to Domon *et al*.

In their studies toward the synthesis of the IJLM-rings portion of the polycyclic ether ciguatoxin CTX3C [51], Domon and co-workers addressed the preparation of the terminal anomeric LM spiroketal (Scheme 20) [52]. Acidic treatment of hemiketal **113** led to a mixture of spiroketals with a 3:1 ratio in favour of the (g,g)-isomer **114**. Working on a more advanced intermediate **116**, spirocyclization was achieved through an oxidative radical pathway to provide a 1:1 mixture of both epimeric spiroketals **117** and **118**. Kinetic control conditions could be assumed for the formation of the (a,g)-spiroketal as conversion to the more stable (g,g)-epimer was obtained after subjection to CSA.

**Scheme 21:**
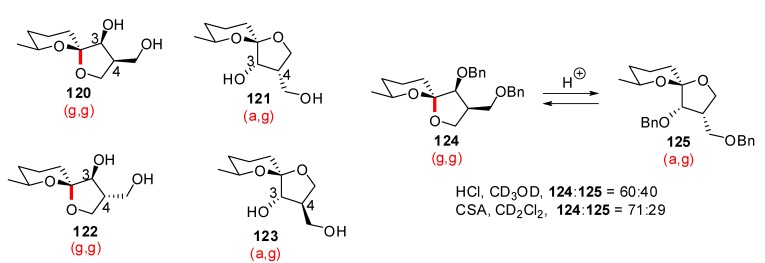
Isolation and epimerization of okaspirodiol according to Beder *et al*.

Okaspirodiol (**120**) was isolated by the group of Beder from *Streptomyces* sp. strain Gö TS 19 as a secondary metabolite and its structure was assigned on the basis of NMR, mass spectroscopy and X-ray analyses, after derivatization (Scheme 21) [53]. The (g,g)-spiroketal **120** was readily isomerized under mild acidic conditions to furnish a set of new structures. After 48 h in deuterated chloroform, 3 new isomers were formed and separated by column chromatography to yield a 29:47:5:19 ratio of **120**:**122**:**121**:**123**. Interestingly, okaspirodiol was not isolated as its thermodynamically favored *trans* (C3, C4) configuration but this structure was the major isomer under acidic conditions. The epimerization at C(3) position was completely prevented after per-benzylation and the equatorial spiroketal was obtained with up to 40% yield, after treatment with methanolic HCl. This increase in the proportion of the non-anomeric (a,g)-spiroketal results from the suppression of hydrogen bonding stabilization between the hydroxymethyl group at C(4) and the O(6) atom. This result underlines the importance of the intramolecular hydrogen bonds in the structural outcome of spiroketal structures and of possible gauche effect between vicinal dioxy substituents, not to mention differential solvent effects.

**Scheme 22:**
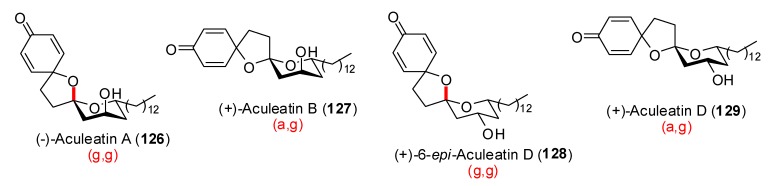
Diverse members of the aculeatins family.

Aculeatins A and B are two epimeric functionalized spiroketals isolated from *Amomum aculeatum Roxb*. [54], together with Aculeatin C [55] and later Aculeatin D [56]. More recently similar structures, the Aculeatols A-D, have been isolated from the same source. These compounds displayed antiprotozoal activity against *Plasmodium* and *Trypanosoma* species as well as antibacterial activity against KB cell lines (Scheme 22) [57].

**Scheme 23:**
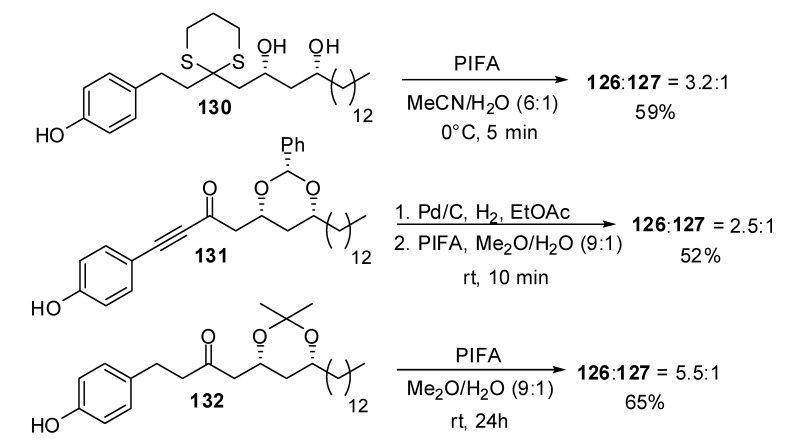
Synthetic approaches to Aculeatins A and B.

Many synthetic efforts have been devoted to the construction of their unusual structure, involving a final key oxidative spirocyclisation of phenolic precursors. In the approach developed by Wong *et al*. [58], rapid treatment of thioacetal **130** with phenyliodine bis(trifluoroacetate) (PIFA) led to a 3.2:1 ratio of epimeric aculeatins A and B, in favor of the (g,g)-spiroketal structure (Scheme 23). Longer reaction times increased the proportion of the (g,g)-spiroketal such as illustrated by the reaction developed by Marco *et al*. [59] on the protected diol **132** wich afforded a 5.5:1 ratio of **126** and **127**. Analogues of **117** were used as starting materials for the preparation of aculeatin D and 6-*epi*-acuelatin D [60]. Starting from enone **131**, hydrogenation followed by treatment with PIFA led to a 2.5:1 ratio of (g,g)- *vs* (a,g)-[6,5]-spiroketals [61].

**Scheme 24:**
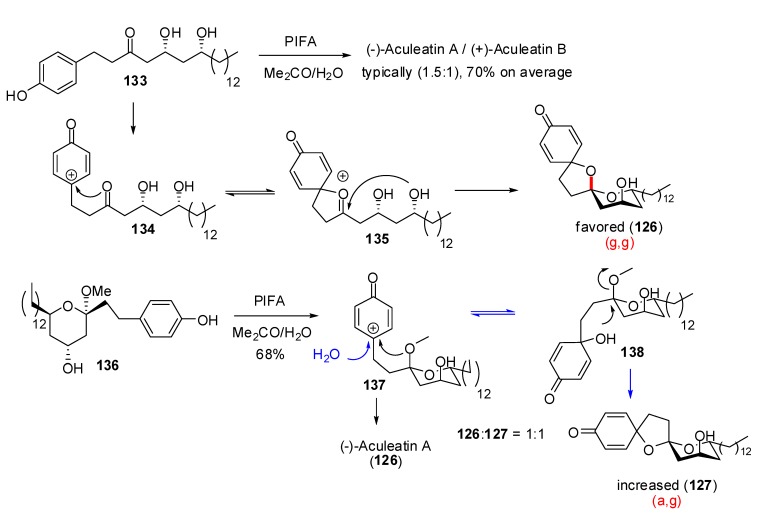
Mechanism of oxidative spirocyclization according to Wong *et al*.

In 2007, Wong *et al*. [62] proposed a rationalization for the stereochemical outcome of the oxidative spirocyclization to aculeatins (Scheme 24). 

The linear precursor **133** led to a mixture of epimers in favor of the anomeric spiroketal **126** through a pathway involving the bicyclic oxycarbenium cation intermediate **135**. Starting from methyl acetal **136**, the authors assumed that the methyl ether was less prone to direct attack on the cation intermediate **137**, thus favoring water addition to produce the tertiary alcohol **138**. Nucleophilic displacement of the methoxy group then led to the (a,g)-spiroketal **127**. The authors developed similar route toward aculeatin D and 6-*epi*-acuelatin D.

**Scheme 25:**
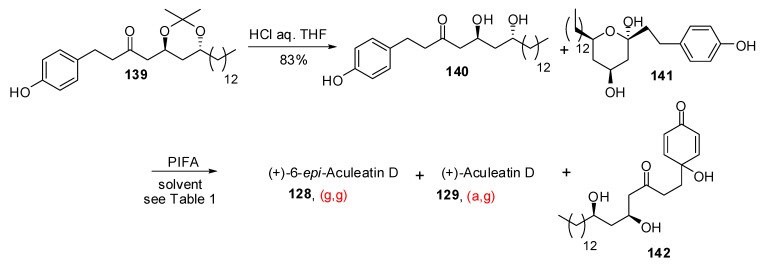
Synthesis of aculeatin D and 6-*epi*-aculeatin D according to Baldwin *et al*.

The group of Baldwin [63] explored the synthesis of aculeatin D from dihydroxy ketone **139** (Scheme 25). Acid mediated removal of the acetonide delivered hemiketal **141** which stands in equilibrium, in solution, with the linear ketone **140**. Further oxidative spirocylization afforded a mixture of (g,g)-spiroketal **128**, (a,g)-spiroketal **129** and open chain derivative **142**, the ratio depending on the solvent. In acetonitrile, only (g,g)-6-*epi*-acuelatin D was formed in 33% yield. In acetone and wet acetone (a,g)- aculeatin D was formed and was isolated in up to 19% yield.

**Table 1:** Stereochemical dependence on reaction conditions.

Conditions	114 (%)	113 (%)	127 (%)
MeCN, 0°C, 1h	0	33	15
MeCN/H_2_O (6:1), 0°C, 1h	0	29	44
Acetone, rt, 20 min	11	40	20
Acetone/H_2_O (9:1), rt, 20 min	19	43	27

**Scheme 26:**
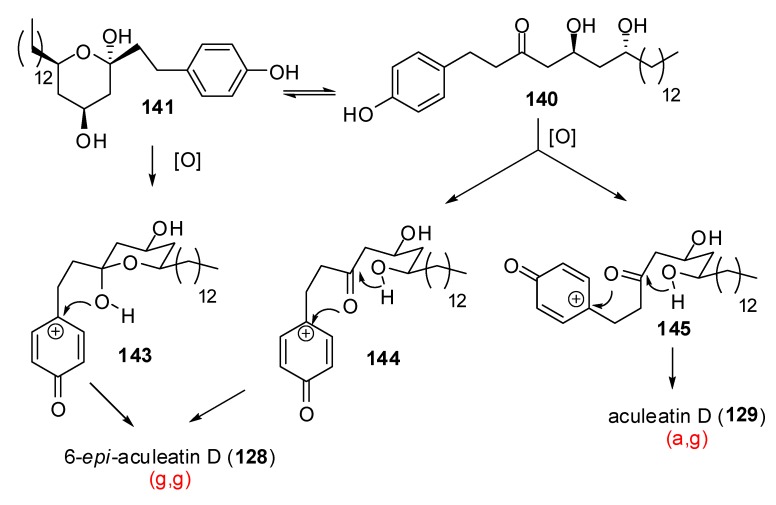
Mechanistic proposal for the oxidative spirocyclization according to Baldwin *et al*.

In acetonitrile, hemiketal intermediate **141** is observed whereas in acetone, a mixture of hemiketal **141** and open chain derivative **140** is observed (Scheme 26). After oxidation, the hemiketal can directly react with the phenoxycarbenium cation **143** to deliver the (g,g)-6-*epi*-aculeatin D, whereas two pathways are possible for the open chain carbocation intermediate giving also access to the expected less stable (a,g)-spiroketal.

## Conclusions

Nature is an infinite reservoir of fascinating compounds of biological interest. A large variety of natural products contain [6,6]-and [6,5]-spiroketals with various configurations that enjoy or not from conformational anomeric stabilization effects for their acetal moiety. Steric factors and gauche effects also contribute to the relative stability of polysubstituted spiroketals. These fragments are very important for the biological activity of compounds that contain them. Many synthetic efforts have thus been devoted to their stereoselective preparation. Whereas the access to the most stable stereoisomers is generally warranted by the use of acidic conditions that permit equilibration of all stereoisomers into the most stable one (ones), the synthesis of less stable stereoisomers is more challenging. It requires stereoselective acetal forming reactions under media that do not allow for acetal heterolysis, or concomitant reactions giving derivatives that can be obtained only from the less stable acetal stereoisomers. This can be realized with adequately substituted systems that can generate tricyclic structures in irreversible reactions.
